# Editorial Notes

**Published:** 1906-03

**Authors:** 


### Editorial Notes.

**Bristol Royal Infirmary.**

Much has been heard of the President's scheme for raising £50,000 for improvements and renovations of this old institution, and the financial statement which he made to the Annual Board on February 14th shows with what energy and success the work has been carried out. The accumulated debt, formed by the deficits of many years, has been wiped out, and the total sum promised or received amounted to £43,577 3s. 8d., leaving a deficiency of only £6,422 16s. 4d. For the first time within the history of the Infirmary in recent years the President was able to report that the ordinary income for the year had exceeded the ordinary expenditure—every single item of receipt had shown an increase and nearly every item of expenditure had decreased accordingly there remained a balance in hand amounting to fifty pounds. We can heartily congratulate the President and the Committee on this result.

**The Sanatorium for Consumptives.**

What may be called a symposium on this subject was published by the *Lancet* in its first number of the new year, and the question is again asked, Do they pay? One cannot but wonder why this question is so often repeated, and why the often repeated answers are not accepted. The writers on this occasion are fairly unanimous in their verdict that the Sanatorium is a neccssary part of the anti-tuberculous crusade, which has already done much to reduce the mortality of the disease, and which may reasonably be expected in the course of some fifty or a hundred years to exterminate the disease altogether. Meanwhile the educational work of the Sanatorium is going on; every patient leaving a well ordered institution of this kind becomes a missionary of good hygiene, and will be able to preach the gospel of good air to those with whom he may be associated.

The following letter from Sir W. H. Broadbent, Bart., who recently paid an official visit to the Winsley Sanatorium on behalf of the National Association, is very gratifying to the Committee which has carried out the establishment of this home for the comsumptive poor:—

84 BROOK STREET, GROSVENOR SQUARE, W.,
*February 5th*, 1906.

MY DEAR DR. WEATHERLY,—Both Dr. Perkins and I were greatly pleased by our visit to the sanatorium. You have solved the problem of efficiency with economy in construction and maintenance, and you had previously set the entire country an example as to the concurrent erection and endowment of such an institution.

The situation is excellent from every point of view, and very beautiful. The architecture of the buildings strikes one as pleasing; and the combination of the two blocks, with the shelters one above the other, is very pretty and picturesque.

The patients' rooms are all that could be desired in the way of cheerfulness, comfort, and free access of air, and I do not know that there is a single improvement I could suggest. I learnt, indeed, a great deal which will be useful to me.

What most astonished me was the small space occupied by the kitchen and its annexes and by the laundry, but the arrangements are evidently good, and experience has shown that the provision for all demands is adequate.

Your sanatorium deserves honourable recognition on every ground.

Yours very sincerely,
W. H. BROADBENT.

**Sanatorium Life.**—The following account of a personal experience of life in the sanatorium for consumptives at Winsley is likely to remove many misconceptions and prejudices of which we frequently hear. The Editor thinks it will be of interest to the readers of this *Journal*, and more particularly to those who have worked so long for the establishment of an institution which is proving to be of great value to the community.

### SIXTEEN WEEKS AT WINSLEY.

### A RECORD OF PERSONAL EXPERIENCE OF THE OPEN-AIR TREATMENT OF CONSUMPTION.

BY A PATIENT.

---

Being run down and feeling unwell, I was advised to consult a specialist, who informed me that my case required complete rest, and advised me to get to a sanatorium.

I pictured in my mind open air with a vengeance, food not of the most appetising nature, raw meat, fat and greasy food which the stomach would turn against, stringent rules, &c.

I made an application to go to Winsley Sanatorium, and in due time I received an intimation that a room was ready for me. Accordingly, taking leave of my friends, I journeyed forth to my new home. One gave me a month, another gave me about ten days to put up with the treatment, as a good many people are under the impression that the rules to be carried out are very stringent, as they might appear to be at first sight.

I arrived at my destination in the afternoon, and was ushered into the reception room. Refreshments were brought me, and after a rest I was presented to the resident medical officer, who, after going through my papers and asking a few questions, dismissed me until the following morning. I was then taken to my new quarters, and was very pleased to find that each patient had a separate room, with every necessary article of furniture, and a lock-up for his own personal belongings.

On my first visit to the dining-room I expected to see a number of patients more or less in a weak state of health, but to my surprise a picture of health and happiness met my eyes—faces with a ruddy complexion almost unknown to the town dweller, and everyone seemed to be on good terms with themselves, happy and contented.

The next day I was given my instructions, and was informed by the doctor that my success greatly depended on my own perseverance. I may say that I had thoroughly made up my mind that it should not be my fault if I did not recover my health. One thing every patient was required to do was to be as lazy as they possibly could be; now, being of an active nature myself, this came a bit hard at first, but like a good many other things, use becomes second nature, so I did not find the time hang as heavily as one might expect with nothing to do.

No doubt a good many would wonder, as I did, what kind of treatment is carried out to meet with the success that this institution is certainly doing. The general treatment, I soon found, was open air, thorough cleanliness, good food and plenty of it. A good many are under the impression that there is a kind of a stuffing process at these institutions similar to the fattening of turkeys, but I did not find this to be the case. You are certainly expected and encouraged to make good meals; but before you are a resident many days you find you have an appetite that would shame a good many animals and surprise yourself. There is also a certain amount of rivalry amongst the patients, each liking to outdo the other; as weighing day comes round this causes an enthusiasm, the consequence of which is that you gain a little weight every week, you are feeling better, and nothing succeeds like success. I will here give a brief outline of the treatment.

*Open Air.*—You are practically in the open the whole of the time. In the daytime you are out in the open, wet or fine; at night when you are in bed you are certainly under cover, but the bedroom windows are very large and of course wide open, so you are in the same temperature whether inside or outside of the building, night and day. The dining-room is on the same principle, windows wide open, therefore you are almost immune from taking cold, in fact a cold is almost an unknown thing amongst the patients. My visit lasted through two of the autumn and two of the winter months, and I was not troubled with a cold, but I contracted one as soon as I came away.

*Baths.*—You are instructed to have a wash from head to foot every morning, warm or cold as the doctor directs. I have known a good many have a cold bath morning after morning, I did myself; this no doubt seems very strange for invalids, and especially in the winter, but I feel sure anyone taking up this morning luxury would be well repaid.

Now comes the most important item—*food.* Well, I must say it takes you a day or two to get used to it, but as I said before you soon do get used to the diet. For breakfast there was a general fare for every day of the week, consisting of porridge, fried bacon, ham, fish, &c., with tea and the usual pint of milk that you were always expected to drink. For dinner there was a change of diet daily, consisting of raw and cooked beef, mutton, pork, steak puddings, a plentiful supply of vegetables, with various kinds of puddings, mostly milk puddings, with the usual quantity of milk. Supper varied—tripe and onions, sausages, fish, puddings, bread and butter, cheese, &c., and the usual quantity of milk. The food generally was well cooked, except of course the raw meat, and this was served up in such a manner that the most delicate stomach could take it. This is only served for a limited period, according to the nature of the illness, then you are put on ordinary diet. The food, I think you will agree with me, is not to be complained of, although we are apt to find grumblers in all classes of society. I suppose it is one of the blessings Nature endowed us with to be critics on everything except that which concerns our own actions.

In a very short time the patients come to know each other, and it is quite possible to find suitable associates, which helps to pass away the time. There are plenty of games such as chess, draughts, cards, &c., and good books to be obtained from the library by those fond of reading. The patients would get up little concerts amongst themselves, and kind friends would visit the sanatorium to entertain us, which all helped to break the monotony of the daily routine of life, in fact I did not find it half so dull as I expected. There are nice walks all around for those who are able to take them, and places of interest to visit in the near neighbourhood. I found the doctor and staff always ready and willing to do any little service for the comfort and welfare of any patient, and there was a marked absence of any partiality. Every patient was personally interviewed once a day at least, and in other cases as often as was necessary. The doctor was a man of keen observation, and very soon read every patient like a book. Your progress was noted from day to day, and your instructions as to exercise, &c., given you.

There were certainly some remarkable cases which came under my own observation during my stay, and it may be of interest to some to know what it is possible to do. In one case a man had been in bed for ten weeks; he was taken out of his bed and brought to the sanatorium, was carried to his room, and of course put to bed. In one week he was up and walking about, in a fortnight he had gained seven pounds in weight, and in one month he could take a walk of two or three miles without being fatigued. Another case was that of a man taken to the institution on his back a good many miles in a conveyance, he had also been in bed several weeks. In one week he was up, and did not require to go to bed again except for his usual rest. He began to put on four and five pounds a week in weight; during four months he gained thirty-two pounds. He left the institution practically a new man and able to go back to his work. Another case, a female patient, gained as much as thirty-three pounds in four and a half months. Of course these are a few exceptional cases, but I could mention several cases where three, four, and five pounds have been gained in one week. On the other hand, I have known others who have not been so fortunate, who require longer treatment than the time that is allowed, which is four months, without special permission; but every patient, I am sure, will learn a lesson they will not forget the remainder of their life—the value of fresh air, cleanliness, &c. I feel certain that a good many like myself feel thankful there is such a place as Winsley Sanatorium—those who are not over blessed with this world's riches, and are unable to pay three or four pounds a week for treatment at a private institution, as I am given to understand you are expected to pay.

I would advise any person who requires to get such treatment, and is fortunate enough to get to Winsley Sanatorium, in a free bed or a maintained bed, to go with a good heart, make up their minds to carry out the instructions as far as lies in their power, and good improvement if not complete success will be their return. I am sure there are no very stringent rules laid down but what are within the reach of the most feeble.

I would advise any and every one who has an opportunity to visit this institution; they will find that they are welcome, and will be shown round to inspect the buildings and grounds, and will have the pleasure of seeing a model sanatorium. I feel sure that if some of our philanthropic friends were to visit and see the good work that is being carried on there would be no lack of subscribers.

Great praise is due to the instigators and managers of this institution, for there appears to be one object in view, and that is to find out the best means of combating this terrible disease, consumption. Of all the good work done and resolutions passed by the Bristol Health Committee none will surpass that of taking the twenty beds at the Winsley Sanatorium for those poor mortals who otherwise may have found an early grave.

I shall look back upon my visit with thankfulness for some time to come, not only for the benefit received, but also for the kindness shown to me, and the many friends I made during my residence. I can assure you that there are worse things than life in a sanatorium.

N.B.—The Editor has received from the medical officer of the sanatorium the following particulars of this case:—

A man, aged 35, admitted on August 29th, 1905, in appearance typically phthisical, with a well-marked hectic flush, with a family history of phthisis, a cough since February, loss of weight, night sweats, and some loss of voice. Physical examination showed evidences of early infiltration at the right apex, and some fibrotic changes at the left: there was retraction under the left clavicle with marked myoidema. Both vocal cords were much thickened and pale. The sputum was fairly characteristic, although the examinations for Tubercle Bacilli gave a negative result. There was evening pyrexia, 99° to 101.5°, during the first few weeks.

After four months' routine treatment he was discharged with no signs of active disease, with a normal temperature, having gained fifteen pounds in weight, and having proved himself capable of performing light work with impunity. On March 7th, 1906, patient was feeling well, with little cough, and no sputum, and was well able to do full work, which he had been doing since leaving the sanatorium. He appeared to be in good health, the disease having been effectively checked.

Dr. Northrup's remarks on living in good air (air de luxe) (*Medical Record*, 1906, lxix., 181) are very appropriate. He remarks: "Our rooms are practically bell jars. . . . The thin, dry man, and his fat, perspiring neighbour sit down together in the bell jar and amiably exchange exhalations, gases, and body emanations. . . . Long experience teaches that close air is not quickly fatal. By air de luxe I do not mean five-dollar canned air made by the hired man. I mean another kind, delivered day and night without extra charge; one that does not smell of rubber nor dry the throat, that does not go to waste in the struggling, nor annoy the dying. It is not one of those physical products controlled by syndicates, pooled by trusts, and cornered by kings. The poor throw it away, and the rich pick it up—in Switzerland. . . . It is air to spare."

An article on clean air by Dr. Mitchell Prudden in the same journal (p. 165), remarks: "Infectious diseases of the respiratory organs are steadily increasing as people are more and more huddled together in offices, dwellings, travelling conveyances, and places of public assemblage. A large part of these diseases are directly traceable to infectious material cast off, in spitting, coughing and sneezing, from the mucous membranes of those suffering from various grades of local disease, and floating for longer or shorter periods in the air as dust or invisible spray."

Sanatorium treatment must inevitably be disappointing if we admit all cases whether incipient or advanced; hence the imperative need for a careful classification of cases before admission, lest a hopelessly advanced case may prevent an incipient one from obtaining the radical cure which he has a right to expect from sanatorium life. It is easy to expect too much, the public commonly does, but the pathological imagination of the doctor should indicate to him the folly of expecting that every case of phthisis is likely to be cured by any sanatorium methods. The finding of suitable employments for patients after the sanatorium course is a difficult one, but it must be admitted that few patients can safely resume their previous occupation without a serious risk of relapse. A paper by Dr. Lucas (p. 12) calls attention to this and other difficulties in the effectual treatment of phthisis amongst the poorer classes, but we hope that the report (shortly to be published) on the first year of working of our own sanatorium at Winsley will show that it is well worth while, both from therapeutic and economic aspects, to do everything possible for the poor; whilst the disease is allowed full scope with them it will be quite useless to deal with it amongst the rich, and all attempts to stamp out the disease will be futile. It must be accepted that these sanatoriums are expensive, and that the public are not very willing to find the money for their equipment, but it is agreed on all hands that the disease cannot be controlled without them, and that the anti-tuberculous crusade must be pushed at all costs if we are to succeed in our efforts to subdue and eradicate this ubiquitous and fatal disease.

**The Proposed University of Bristol.**

If one may judge from the applause with which references to the foundation of a University are invariably greeted, it would appear that a practical scheme for realising the idea would at once meet with enthusiastic support. It is, however, no less obvious that until some responsible body puts forward such a scheme, makes it clear that there is a need for a University in Bristol, and establishes the principles of its constitution further progress is impossible.

It is, perhaps, not too much to say that the term "University" has little meaning to the average individual. Some connect it only with Oxford and Cambridge, others think of a University as an examining body, like the London University on its external side, while many have heard of the Universities which have recently been established in Liverpool, Manchester, Leeds, Birmingham, and Sheffield, and, without understanding the details of their constitution, think that Bristol, as the next greatest city, should follow their lead. In this there is not only natural pride, but good sense; for as these Universities are established on the same principles, and have almost indentical constitutions, it is more than likely that the foundation of a similar University in Bristol will be to the advantage of the city.

A University should not be in any sense merely an examining body. Its function is to decrease the sum total of human ignorance, and to teach its students to take part in this work. Those occupations known as the "learned professions"—the church, medicine, chemistry, law, engineering, &c.—involve the solution of problems of a more or less difficult character. Efficiency in these professions demands not only a wide knowledge of the facts and of the theories relating to them, and in some cases a certain manipulative skill, but the power of examining and collating facts as they appear, and of interpreting the results.

Training for such professions should then involve as an essential feature a course of study under teachers who are themselves investigators, and who have these principles always in view. The courses of study should be arranged so as to give the student the best possible training for the profession he has in view, and the examinations should be of such a character as to test his attainment at the end of the course. It must be clearly understood that the course of study is the essential, and the examination, which is conducted by the external examiner and the teacher jointly, is subordinate to it. Degrees awarded on this basis carry weight according to the reputations of the teachers; they are fast attaining a far higher value than the "external" degrees, which, except in medicine, demand no course of study and encourage cramming.

Before Bristol can apply for a University charter facilities for University education in the city must be extended and organised so as to avoid wasteful competition. Before this can be brought about the meaning of the term "university education" must be clearly established. No body of men more clearly understands the value of sound professional training, designed with a view to increasing the powers of observation and inquiry, and directed by men who are themselves experts and specialists, than do the members of the medical profession. The extension of this principle to other departments, and the elimination of the cramping influence of external examining bodies, will only be possible under the University, and it is this fact that the medical profession may help to keep in view.

There is no reason why the Medical School of the University should not be as strong as any other in the country. The Faculty of Science will be able to provide for preliminary medical studies in the departments of chemistry, physics, zoology and botany in a manner equal to any other provincial school. Further provision will have to be made for the teaching of intermediate medical subjects, and sufficient funds should be forthcoming to properly equip and endow such departments as those of anatomy and physiology. It is very essential, both for the sake of students who are following the regular course, post-graduate students, and men in practice in the city and district, that ample provision should be made for study and research in such subjects as pathology, bacteriology and the more specialised and scientific subjects allied to medicine. The establishment of lectureships and fellowships and the foundation of properly-equipped laboratories will make it possible to utilise the vast quantity of clinical material which is available.

The details of a scheme for the organisation of the Medical Faculty of the University can of course only be arrived at after careful inquiry.

**The Royal Sanitary Institute.**

THE Annual Congress of this Institute will be held in Bristol in July. The preparations for this meeting are well advanced. At a meeting held in the

Guildhall on February 2nd, under the chairmanship of the Lord Mayor of Bristol, an Executive Committee was appointed, and it was announced that the Right Hon. Sir Edward Fry, P.C., had accepted the office of President. A very full programme of meetings and receptions is arranged, and a Health Exhibition will be held in the Drill Hall from July 9th to 21st. The sanitary officers of the city and many members of the Corporation are taking a warm interest in the meeting, which is likely to be quite as successful as congresses usually are in Bristol.

**The National League for Physical Education and Improvement.**

A Bristol Branch of this League was inaugurated at a large meeting in the Colston Hall, held on February 12th, 1906, under the presidency of the Lord Bishop of Bristol. The objects of the League were stated in our last number, and have since been further formulated as follows:—

1. To organise all power working for racial improvement, and concentrate the now scattered forces contending against such moral and social evils as Overcrowding, Infant Mortality, Intemperance, &c., which all imperil the future of the race and the stability of our nation.

2. To establish a National or Central Institute for Physical Education with a Training College and Examining Body—to draw into this much-needed work men and women of the educated classes.

3. To arrange the physical examination of school children, with remedial opportunities where defect is present.

4. To spread knowledge of the common laws of health by means of lectures and the dissemination of literature.

This meeting was largely attended, the large hall being well filled, and the audience appeared to be much interested in the subject as it was put before them by the various speakers, who had come to represent the central organisation of the National League for the purpose of inaugurating the first of many branches which are to be formed in all parts of the country. The Central Council consists of a very large number of wellknown persons who have taken an interest in the subject, and the Executive Council intends to make the League a power in the land.

The speakers on this occasion were The Right Hon. Sir John Gorst, P.C., Sir Lauder Brunton, M.D., D.Sc., F.R.S., Mrs. Bramwell Booth, Major-Gen. Sir Fred. Maurice, K.C.B.

At the end of the meeting it was resolved on the proposition of Professor Lloyd Morgan, supported by Judge Austin and Dr. R. Shingleton Smith, "That a Bristol branch of the National League for physical education and improvement be formed, with the Lord Bishop as President, and a committee composed of those present by invitation on the platform, and others who have signified their consent, with power to add to their number." The Lord Mayor expressed his hearty sympathy with the objects of the meeting, and trusted that everything possible would be done to promote the success of the League.

Sir Lauder Brunton, in pointing out the objects of the League, spoke of the deplorable and unnecessary infant mortality, far exceeding the massacre of the innocents at Bethlehem by King Herod, and he added that in order to stop this waste of human life and energy a great deal could be done by proper physical training. For this purpose there must be some national central institute for physical education, with a trained examining body, which must have authority to issue diplomas, without which no person should be allowed to teach. In this way unity would be established throughout the country, and we should get the best possible training in every part of it. The establishment of such an institute was proposed by Miss Theodora Johnson in November, 1904, and the proposal was now beginning to take definite shape; but all children were not alike, and the physical exercise that was barely enough for one was enough to exhaust and injure others, and in cases of which he had known the heart had been seriously damaged by over-strain. It was, therefore, absolutely necessary that there should be an examination into the physical condition of children, so as to ascertain how much they could do with advantage and without injury. All the children should be made to learn the general laws of health, the evils of spitting, the use of fresh air with avoidance of chill, the care of the teeth, and the mischief that may be done by tobacco and alcohol. The improvement of the health and physique of the nation was a very complex problem. It needed the co-operation of everyone, and especially it required the co-ordination of all bodies already working to attain the end. Many of these bodies were not even known to one another, and in order to ensure their co-operation a National League for Physical Education and Improvement had been founded. They hoped to save the babies, to help the children, to train the youths, to raise up a race of sturdy men and women, and to lessen the number of the diseased, the feeble, the unemployed, and the criminals.

Dr. Shingleton Smith pointed out that the self-denying ordinance of the abolition of disease and the improvement in the health of the people had been the glory of the medical profession of the past. Much had been already done. Even within recent years we all could remember the abolition of typhus, the almost complete extinction of small-pox, the control of malarial fever, and the gradual diminution of the death-rate from tuberculous diseases; these facts showed that the ultimate control and elimination of many diseases are not Utopian dreams of the enthusiast. So much has the mortality of phthisis been reduced during the last two or three decades, that we may reasonably look forward to the not far distant future when the tubercle bacillus may be as extinct as the dodo. The medical profession could not do otherwise than favour in every way possible the functions of the league proposed, as it showed that they would have the assistance of the State, the Church, and the public in supporting their endeavours to improve the health and the physique of the nation.

